# *Porphyromonas gingivalis* Strain Specific Interactions with Human Coronary Artery Endothelial Cells: A Comparative Study

**DOI:** 10.1371/journal.pone.0052606

**Published:** 2012-12-26

**Authors:** Paulo H. Rodrigues, Leticia Reyes, Amandeep S. Chadda, Myriam Bélanger, Shannon M. Wallet, Debra Akin, William Dunn, Ann Progulske-Fox

**Affiliations:** 1 Department of Oral Biology, College of Dentistry and Center for Molecular Microbiology, University of Florida, Gainesville, Florida, United States of America; 2 Department of Anatomy and Cell Biology, College of Medicine, University of Florida, Gainesville, Florida, United States of America; 3 Department of Periodontology, College of Dentistry, University of Florida, Gainesville, Florida, United States of America; INSERM, France

## Abstract

Both epidemiologic and experimental findings suggest that infection with *Porphyromonas gingivalis* exacerbates progression of atherosclerosis. As *P. gingivalis* exhibits significant strain variation, it is reasonable that different strains possess different capabilities and/or mechanisms by which they promote atherosclerosis. Using *P. gingivalis* strains that have been previously evaluated in the *ApoE* null atherosclerosis model, we assessed the ability of W83, A7436, 381, and 33277 to adhere, invade, and persist in human coronary artery endothelial (HCAE) cells. W83 and 381 displayed an equivalent ability to adhere to HCAE cells, which was significantly greater than both A7436 and 33277 (*P*<0.01). W83, 381, and 33277 were more invasive than A7436 (*P*<0.0001). However, only W83 and A7436 were able to remain viable up to 48 hours in HCAE cell cultures, whereas 381 was cleared by 48 hours and 33277 was cleared by 24 hours. These differences in persistence were in part due to strain specific differences in intracellular trafficking. Both W83 and 381 trafficked through the autophagic pathway, but not A7436 or 33277. Internalized 381 was the only strain that was dependent upon the autophagic pathway for its survival. Finally, we assessed the efficacy of these strains to activate HCAE cells as defined by production of IL-6, IL-8, IL-12p40, MCP-1, RANTES, TNF-α, and soluble adhesion molecules (sICAM-1, sVCAM-1, and sE-selectin). Only moderate inflammation was observed in cells infected with either W83 or A7436, whereas cells infected with 381 exhibited the most profound inflammation, followed by cells infected with 33277. These results demonstrate that virulence mechanisms among different *P. gingivalis* strains are varied and that pathogenic mechanisms identified for one strain are not necessarily applicable to other strains.

## Introduction

The Gram-negative anaerobic periodontal pathogen, *Porphyromonas gingivalis* is gaining recognition as a contributor to cardiovascular diseases such as aortic aneurysm [1], [2] and atherosclerosis [3], [4]. Spontaneous *P. gingivalis* bacteremia has been reported in patients with low grade periodontal disease, and the frequency of detection of the pathogen in the blood or in circulating dendritic cells increases after periodontal treatment [5], [6]. Moreover, several independent studies have detected *P. gingivalis* DNA or retrieved live bacteria from human aortic aneurysms, aortic thrombi, atheromas, and atherosclerotic plaque specimens [1], [2], [7]–[15]. Experimental infection with different strains of *P. gingivalis* have shown that the bacterium can promote varying degrees of cardiovascular disease including endothelial dysfunction [16], vascular smooth muscle proliferation [17], [18], aortic aneurysm [19]–[21], and atherosclerosis [22]–[27]. Although not specific to *P. gingivalis*, intensive periodontal therapy has been shown to improve endothelial function in the long-term in patients with periodontal disease [28].

It has been proposed that periodontal bacteria can promote cardiovascular disease through several mechanisms such as endothelial dysfunction brought upon by circulating pro-inflammatory mediators released from periodontal tissues, endothelial activation as a result of contact with periodontal bacteria or bacterial products, and direct invasion of cardiovascular cells by periodontal bacteria [3], [4]. *P. gingivalis* in particular, can invade various types of endothelial cells [29]–[31], and promote pro-atherogenic responses in these cells including production of pro-inflammatory mediators, increased expression of cell adhesion molecules [32]–[35], and induction of autophagy [36] or apoptosis [37]. With regard to atherosclerosis, experimental studies were limited to the use of fimbriae-expressing strains [25], [27], [38], [39], which demonstrated that fimbriae expression and possibly fimbriae type are major determinants for the pro-atherogenic effects of *P. gingivalis* [39], [40]. More recent work suggests that other virulence factors of *P. gingivalis* may be equally important for the progression of atherosclerosis since the fimbriae-deficient strain W83 [41] also promotes atherosclerosis in *ApoE* deficient mice [26]. In contrast, strain 33277, which is closely related to strain 381 [42] does not accelerate atherosclerosis in *ApoE* deficient mice [16].

Endothelial injury, with subsequent endothelial dysfunction is an early event in the pathogenesis of atherosclerosis [43], which involves ongoing leukocyte and vascular cell interactions that are triggered by repeated metabolic, infectious, or mechanical injuries to the vessel wall. Features of endothelial dysfunction include production of pro-inflammatory cytokines and chemokines, as well as the enhanced expression of cell adhesion molecules that recruit leukocytes into the affected area [44]. Increased autophagy, which can represent an adaptive response of the cell to metabolic stress or inflammation [45], is another characteristic of endothelial dysfunction. Since endothelial cells are among the primary cells to be affected during atherosclerosis, these have been used extensively as an *in vitro* model system for identifying potential virulence mechanisms of *P. gingivalis* [29], [34], [36, [46]–[49].

Our objective for this study was to perform a comparative study between *P. gingivalis* strains W83, A7436, 381, and 33277 since these strains produce varying disease outcomes in ApoE deficient mice (Table 1) [16], [26], [27], [50]. Moreover, these strains also express a different array of virulence factors that may impact microbial/endothelial cell interactions such as fimbriae type (Figure S1) [51], fimbriae expression [52], [53], and polysaccharide capsule type [54] (Table 1). In this study we compared the ability of these strains to adhere, invade, and persist in human coronary artery endothelial cells (HCAEC), which are a relevant endothelial cell type for atherosclerosis [34]. We also assessed HCAE cell responses to W83, A7436, 381, and 33277 by measuring their production of pro-inflammatory cytokines, chemokines, and soluble cell adhesion molecules [32]–[35]. We were able to demonstrate that these four strains of *P. gingivalis* exhibit diverse abilities to 1) invade and persist in endothelial cells, 2) traffic within these cells, and 3) induce potentially pro-atherogenic host responses in HCAE cells. Our results suggest that the mechanisms by which *P. gingivalis* promotes atherosclerosis are diverse and strain specific.

**Table 1:** Comparison of *P. gingivalis* strains.

Phenotype	W83	A7436	381	33277
Capsule [54]	Yes (K1)	Yes (K1)	No (K−)	No (K−)
Fim A genotype [52]	IV	IVa)	I	I
Express fimA [41], [52], [61]	No	Yes	Yes	Yes
Accelerate atherosclerosis [16], [24], [26], [39]	Yes	Yes	Yes	No

a) See supporting file Figure S1.

## Results

### Adherence, invasion, and persistence in HCAE cells

**Figure 1:**
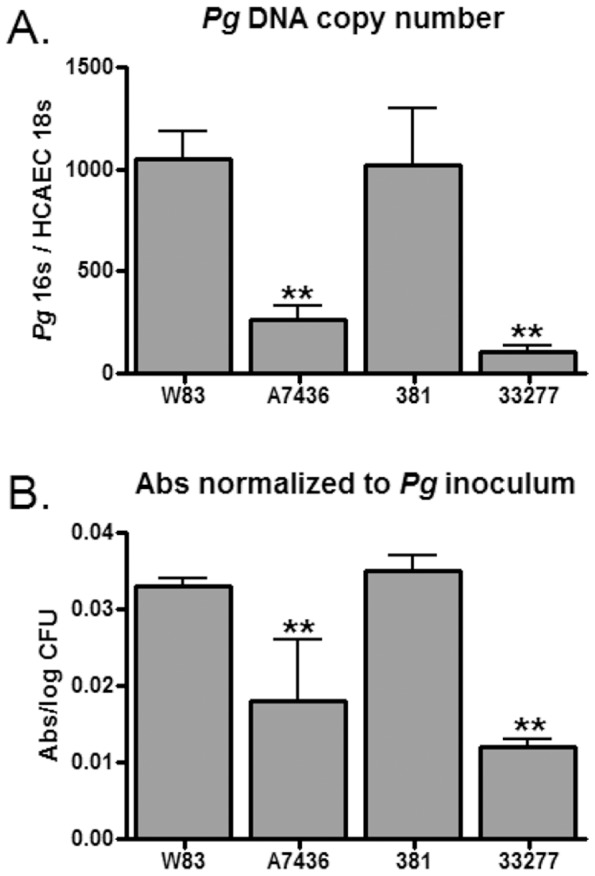
Adherence of *P. gingivalis* to HCAE cells detected by qPCR (A) and ELISA (B). Values represent the mean ± SD of six biological replicates from two independent experiments. Inoculated HCAE cells were incubated at 4°C for 30 min without agitation. (A) *P. gingivalis* 16S DNA copy number was normalized to HCAE cell 18S copy number. (B) Absorbance values at 450 nm for each replicate were normalized to their corresponding inoculums. **Values were significantly less than W83 and 381 (*P*<0.0001).

Since a major determinant of atherosclerosis caused by *P. gingivalis* 381 is invasion of host cells [39], [55], we compared the ability of these strains to adhere, invade, and persist in HCAE cells. Adherence to HCAE cells was assessed by *P. gingivalis* DNA copy number and detection of adherent bacteria by ELISA (Figure 1A and 1B). Both W83 and 381 adhered to HCAEC with equivalent efficacy, which was significantly greater than either A7436 or 33277 (*P*<0.0001).

**Figure 2:**
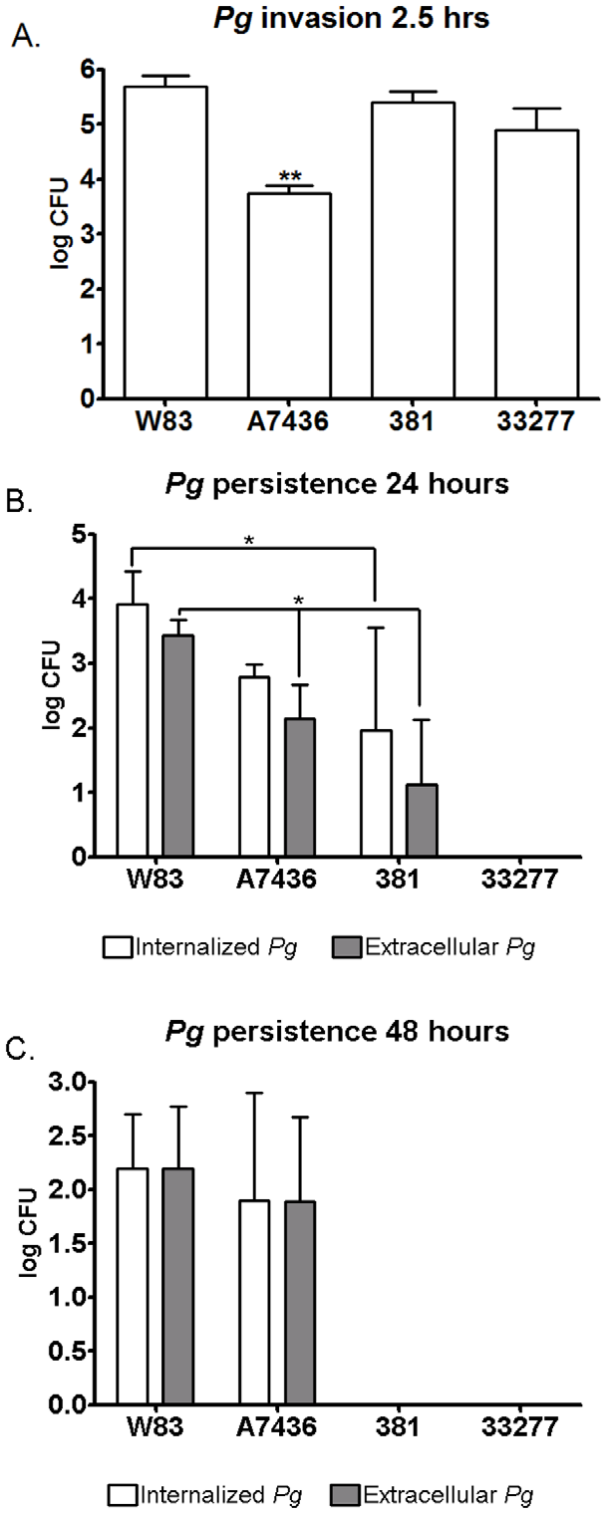
Invasion and persistence of *P. gingivalis* in HCAE cells. Values represent mean log CFU ± SD of six biological replicates from two independent experiments. **Values were significantly different from other groups (*P*<0.0001). * Values were significantly different (*P*<0.05).

We next assessed microbial invasion and persistence in HCAE cells by measuring the number of viable internalized bacteria at 2.5, 24, and 48 hours post-inoculation (Figure 2A–C, respectively). The degree of invasion was determined at 2.5 hours post-inoculation since this was the time point at which the highest numbers of internalized *P. gingivalis* have been obtained with our inoculation method and any extracellular bacteria that were still present within the culture media would be killed by pulse antibiotic treatment [31].

We found the degree of invasion was different among all the *P. gingivalis* strains in that there was a statistically significant but subtle difference between W83, 381, and 33277. Specifically, W83 exhibited the highest degree of invasion, followed by 381 and 33277 (*P*<0.01). The most striking observation was the low numbers of A7436 that were retrieved from HCAEC lysates at 2.5 hours post inoculation (*P*<0. 0001). The pattern of microbial persistence was also markedly different among the strains. At 24 hours post-inoculation, W83 infected cultures yielded the highest number of viable bacteria in both intracellular and extracellular compartments (*P*<0.03). Endothelial cells infected with A7436 or 381 had equivalent numbers of internalized bacteria, but significantly fewer 381 were cultured from extracellular fractions (*P*<0.01). Strain 33277 was not recovered from either the intracellular or the extracellular fraction at 24 or 48 hours post-inoculation. By 48 hours, only HCAE cell cultures that were inoculated with W83 and A7436 yielded viable bacteria, and both strains exhibited equivalent microbial loads in both intracellular and extracellular compartments (Figure 2C).

### Intracellular trafficking of *P. gingivalis*

**Figure 3:**
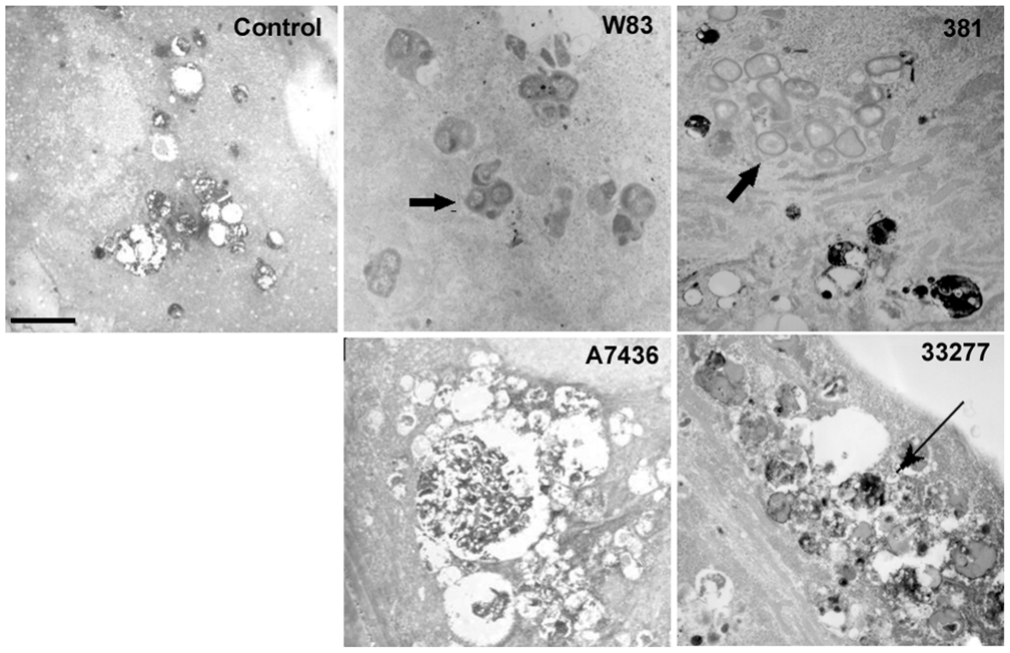
Ultrastructural evaluation of internalized *P. gingivalis* at 6 hours post-inoculation. Transmission electron microscopic images (6000× magnification) of uninfected (control) and infected cells are representative of 3 independent experiments. Block arrows indicate internalized bacteria in CMPase negative vacuoles. Thin arrow depicts bacteria undergoing degradation within CMPase positive vacuoles. Scale bar represents 2 µm.

**Figure 4:**
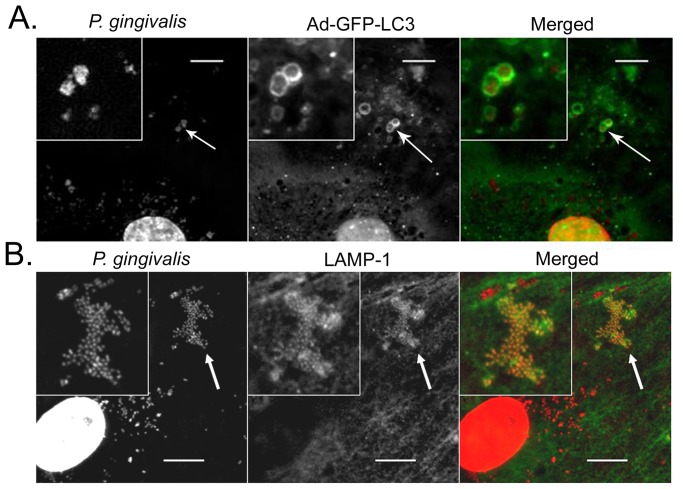
Representative microscopic images of *P. gingivalis* strain W83 within LC3 positive or LAMP-1 positive vacuoles at 6 hours post-inoculation. Arrows indicate magnified inserts. Scale bar is equivalent to 10 µm. Representative images of other *P. gingivalis* strains can be viewed in supporting file Figure S4.

**Figure 5:**
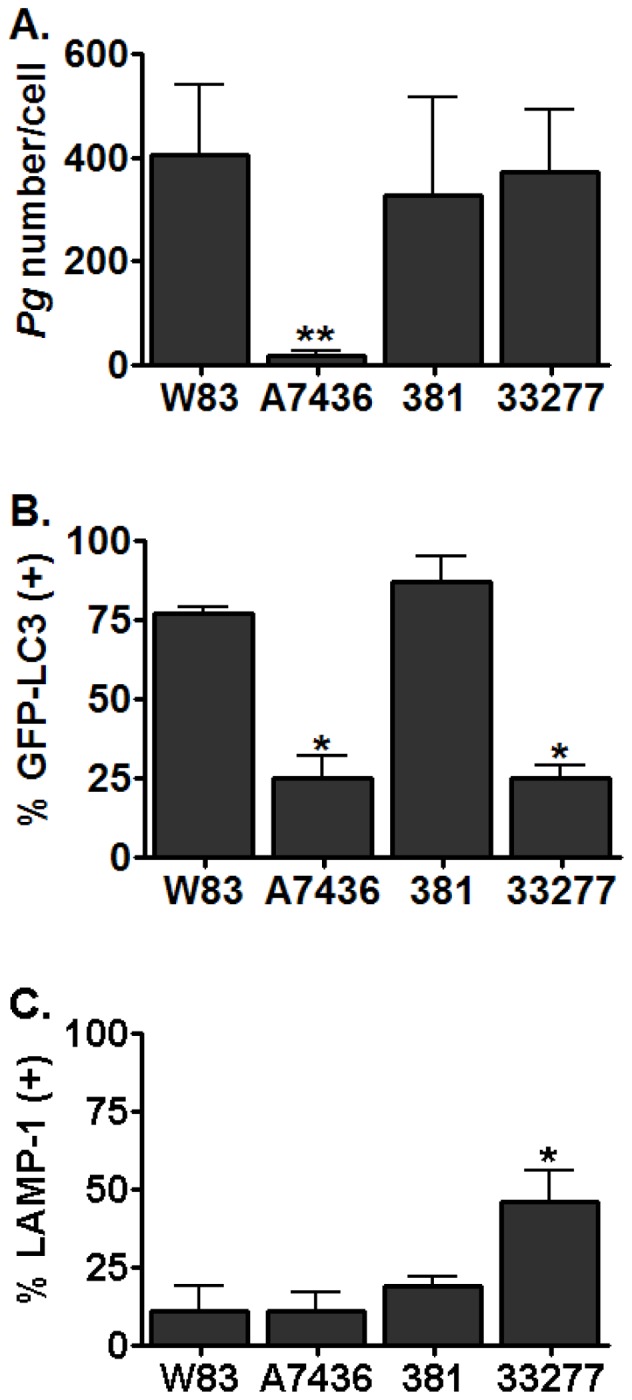
Number of internalized *P. gingivalis* in HCAE cells and their distribution in LC3 or LAMP-1 positive vacuoles. A) Mean number ± SD of internalized bacteria. **Significantly less than other strains (*P*<0.0001). B) Mean percent ± SD bacteria in LC3 positive vacuoles. *Significantly different from W83 and 381 (*P*<0.01). C) Mean percent ± SD bacteria in LAMP-1 positive vacuoles. *Significantly different from other strains (*P*<0.02). All values were obtained from samples collected at 6 h post inoculation and represent three biological replicates obtained from three independent experiments.

In order to determine if the variability we observed in invasion assays was due to differences in intracellular trafficking, we first examined the cellular ultrastructure of infected cells by transmission electron microscopy (Figure 3). Based on previous studies [36], [56], we selected 6 hours as a post-inoculation time point that would most likely detect whether or not internalized *P. gingivalis* were being degraded within lysosomal compartments. Abundant W83 and 381 organisms were observed intact within electron dense vacuoles that resembled autophagosomes [36] whereas 33277 bacteria were observed undergoing degradation. Interestingly, HCAE cell cultures that were infected with A7436 displayed numerous vacuoles full of cytoplasmic ground substance that exceeded what was observed in uninfected cells. It could not be discerned whether these vacuoles contained cellular material and/or degraded bacteria and few internalized A7436 could be located in these cells. We next quantified the total number of internalized bacteria within HCAE cells and their distribution in autophagosomes or endosome/lysosomes at 6 hours post-inoculation. We used the microtubule-associated protein1 light chain 3 (LC3) as a specific marker for autophagosomes [57] and LAMP-1 as a specific marker for late endosome/lysosomes [58] (Figure 4, Figures S2, S3, and S4). There were dramatically fewer A7436 found within HCAE cells when compared to the other strains (*P*<0.001, Figure 5A), and the majority of internalized A7436 were not found in either LC3 or LAMP-1 positive vacuoles (Figure 5B and 5C). Although there were few internalized bacteria found in A7436 infected cells, these cells did exhibit more LC3 positive vacuoles than uninfected fed cells or cells infected with 33277 (Figure S3). In contrast, the majority of W83 and 381 (75% or more) were found within LC3 positive vacuoles (*P*<0.01), whereas only 25% or less of these strains were found within LAMP-1 positive vacuoles. Strain 33277 was unique in that more of this strain was found in LAMP-1 positive vacuoles when compared to the other strains (*P*<0.05).

**Figure 6:**
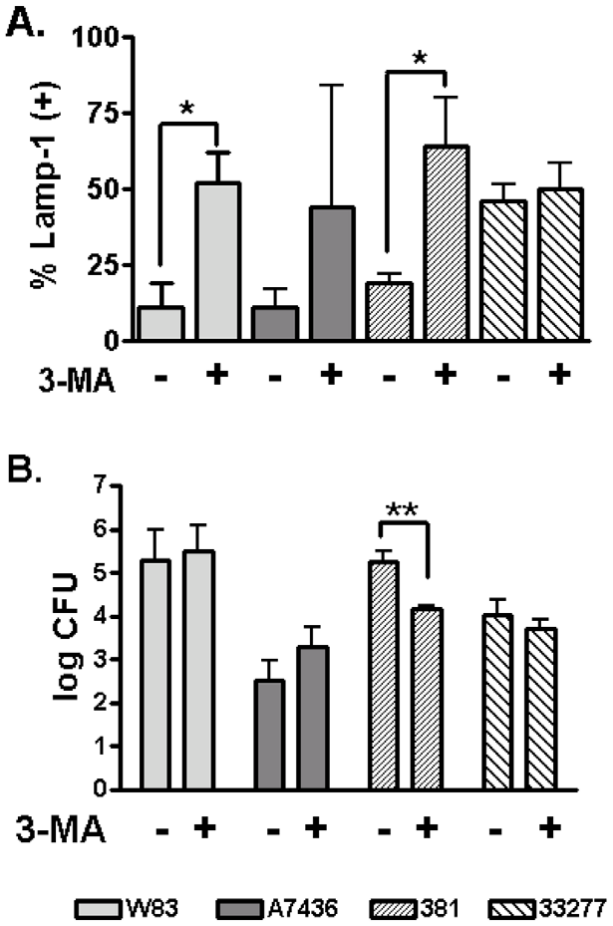
Impact of 3-MA treatment on the intracellular trafficking and survival of *P. gingivalis*. A) Mean percent ± SD of internalized bacteria found in LAMP-1 positive vacuoles obtained from 3 biological replicates from 3 independent experiments. *Values were significantly different (*P*<0.05). B) Mean log CFU ± SD (n = 5) of internalized *P. gingivalis* recovered from HCAE cell lysates at 6 h post-inoculation. ** Values were significantly different (*P*<0.02).

Inhibition of autophagy by 3-methyladenine (3-MA) or wortmannin results in the shuttling of *P. gingivalis* 381 into the phagocytic pathway, where it is killed [36]. Therefore, we evaluated the impact of 3-MA treatment on the intracellular trafficking and viability of W83, 381, A7436, and 33277 strains under normal invasion conditions and after inhibition of autophagy with 3-MA (Figure 6). The efficacy of 3-MA treatment was assessed by lack of LC3 positive vacuoles in *P. gingivalis* infected cells (Figure S5).Treatment with 3-MA resulted in a significant shift of W83 and 381 into LAMP-1 positive vacuoles (*P*<0.05) but not 33277 (Figure 6A). Although 3-MA treatment produced a trend of more A7436 in LAMP-1 positive vacuoles, the effect was variable and inconsistent. As previously reported [36] 3-MA treatment significantly reduced the number of viable 381 retrieved from HCAE cells at 6 hours post inoculation (*P*<0.02). Surprisingly, inhibition of autophagy did not affect the intracellular survival of W83, despite the shuttling of more bacteria into the late endosome/lysosome compartment. Inhibition of autophagy also did not affect the survival of A7436 or 33277 at 6 hours post inoculation.

### Assessment of endothelial activation or dysfunction in *P. gingivalis* infected HCAE cells

**Figure 7:**
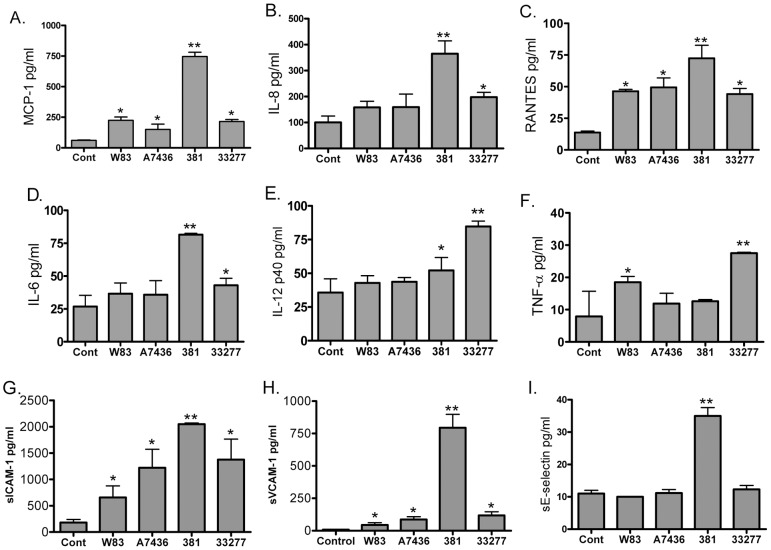
Endothelial response profile to *P. gingivalis* infection at 24 hours post-inoculation. Values represent the mean ± SD of 3 biological replicates from 3 independent experiments. **Values were significantly different from all other groups (*P*<0.05). *Values were significantly different from control (*P*<0.05).

Endothelial cytokine and chemokine secretion is an essential step in the initiation and progression of atherosclerosis [59], [60]. Therefore, we measured cytokines and chemokines that have been shown to be elevated in response to *in vivo* and *in vitro* infection with *P. gingivalis* [59] (Figure 7). In order to allow for sufficient expression of these factors [34], [61], supernatants from HCAE cells were evaluated at 24 hours post-inoculation. Endothelial cells infected with 381 displayed the most pronounced inflammatory response in that these cells expressed the greatest repertoire of pro-inflammatory mediators and soluble cell adhesion molecules. Specifically, HCAE cells infected with strain 381 produced the greatest amounts of MCP-1, IL-8, RANTES, and IL-6 when compared to uninfected cells (control) and cells infected with other *P. gingivalis* strains (Figure 7A–F, *P*<0.05). While cells infected with 381 produced more IL-12p40 than uninfected cells, it was less than cells infected with strain 33277 (P<0.05). HCAE cells infected with strain 33277 produced the highest levels of TNF-α, and more MCP-1, IL-8, RANTES, and IL-6 than uninfected HCAE cells (*P*<0.05). HCAE cells infected with W83 produced more MCP-1, RANTES, and TNF-α than uninfected cells, which was the same amount or less than cells infected with 381 or 33277 (*P*<0.05). Cells infected with A7436 produced the smallest inflammatory response; these cells produced more MCP-1 and RANTES than uninfected cells (*P*<0.05), but the amount was less than cells infected with 381.

Although all strains of *P. gingivalis* induced a significant release of sICAM-1 and sVCAM-1 from HCAE cells (Figure 7G–I), only 381 induced a significant release of sE-selectin from these cells (P<0.05). Since elevated sE-selectin may be an indicator of endothelial injury [62], we assessed the degree of HCAE cell death at 24 and 48 hours post inoculation. Cell viability was assessed by intracellular esterase activity using conversion of calcein AM to fluorescent calcein, and loss of plasma membrane integrity was evaluated by entry of ethidium homodimer-1 into HCAE cells [63]. There was no difference in the percent cell death among uninfected and infected cell cultures, which exhibited less than 10% cell death (Figure S6).

## Discussion

In order to be able to make inferences between the strain specific atherogenic potential of *P. gingivalis*, we limited our study to strains that have already been evaluated in the same atherosclerosis model (the *ApoE* null mouse) using similar experimental conditions [25], [26], [39], [50], [55]. Although our approach did not include a wide range of *P. gingivalis* strains that have been implicated in cardiovascular disease [17], [19]–[21], it did identify unique microbial strain specific interactions with endothelial cells (Table 2). For example, we noted that W83 was highly invasive despite the presence of capsule [54] and the lack of fimbriae expression [41]. This is in contrast to A7436, which also expresses capsule that can reduce its invasive ability [29]. In addition, the *P. gingivalis* strains that were used in our study trafficked differently within HCAE cells, exhibited different abilities to persist in HCAE cell cultures, and elicited different endothelial activation profiles which may be important in the pathogenesis of cardiovascular disease [3], [31].

**Table 2:** Summary of *P. gingivalis* strain specific effects on HCAE cells.

Characteristic	W83	A7436	381	33277
Adherent	++++	++	++++	++
Invasive	++++	++	++++	++++
Persistent	+++	+++	++	−
Induction of autophagy	++++	++	++++	+
Traffic through autophagic pathway	++++	−	++++	−
Inflammatory	++	+	++++	+++
Induce endothelial stress/dysfunction	++	++	+++	++

*P. gingivalis* may contribute to cardiovascular disease through invasion of endothelial cells, which could then perturb host cell processes as well as provide a protective niche for the microbe against host clearance mechanisms [3], [64]. Several studies have shown that *P. gingivalis* strains W83, A7436, 381, and 33277 enter endothelial cells through the endocytic pathway [29], [36], [56]. However, following internalization, *P. gingivalis* strains may be sorted into different vacuolar compartments including late endosome/lysosomes [56], autophagosomes [36], or Rab 11 and RalA positive recycling endosomes through which bacteria can exit the host cell [65]. Trafficking of *P. gingivalis* through the recycling pathway in HCAE cells has not been confirmed, but *P. gingivalis* does exit from endothelial cells and exited bacteria have been shown to enter smooth muscle cells [31]. In our study we also observed exiting of 381, W83 and A7436 from HCAE cells after pulse treatment with antibiotics, and evidence of microbial exit from host cells correlated with microbial persistence in HCAE cell cultures. A similar phenomenon would likely contribute to atherosclerosis *in vivo* by supporting microbial persistence within the vessel wall and spread to other cell types within the vascular intima.

The pathway through which *P. gingivalis* strains traffic within HCAE cells may, in part, be influenced both by microbial load (MOI) and strain specific characteristics (invasive capacity and capsule production). For example, at an MOI of 100, highly invasive 381 primarily traffics through the autophagic pathway [36], but at an MOI of 1000, it is found primarily within the late endosome/lysosome compartment [56]. Using an MOI of 100, we found that both W83 and 381 primarily trafficked through the autophagic pathway, but unlike 381, the intracellular survival of W83 was not dependent upon the autophagic pathway. Since the presence of capsule can defer late endosome/lysosome fusion, this may account for the prolonged survival of W83 in LAMP-1 positive vacuoles during inhibition of autophagy [66]. Although 381 and 33277 are closely related according to heteroduplex and ISR sequence analysis [42], 33277 did not predominantly traffic through the autophagic pathway, even at an MOI of 100. Instead most internalized 33277 were found within LAMP-1 positive vacuoles, suggesting that most of the internalized 33277 traffic from the early endosome to the endosome/lysosome compartment. This may account for the poor survival of internalized 33277, which was observed by TEM to be undergoing degradation. Interestingly, very few A7436 were found within autophagosomes or LAMP-1 positive vacuoles despite activation of autophagy. This suggests that internalized A7436 does not primarily sort into late endosome/lysosomes or autophagosomes. Instead, A7436 may be primarily sorting into the endocyte recycling pathway since nearly 50% of A7436 that were isolated from HCAE cell cultures had exited from host cells.

Microbial infection can promote endothelial dysfunction through various mechanisms [43], [67]. Strain 381 promotes endothelial activation, and possibly dysfunction, through activation of innate immunity and inflammation [32], [34], [39]. Our data were consistent with previous reports in that strains 381 and 33277 induced the greatest degree of endothelial activation, which was probably modulated by fimbriae [33], [34]. In contrast, A7436, which also expresses fimbriae, induced the weakest inflammatory response in HCAE cells. Since we did not include fimbriae deficient mutants in our study, we could not ascertain if this attenuated response is related to a difference in fimA genotype or fimbriae expression. Capsule expression by A7436 as well as W83 could explain the attenuated inflammatory response observed in cells infected with these strains. Both A7436 and W83 are K1 positive, and capsule expression by *P. gingivalis* has been shown to reduce the inflammatory response in the host [68], [69].

Since HCAE cells infected with W83 or A7436 did not exhibit profound activation compared to cells infected with 381, it is possible that innate activation is not as dominant a factor in atherosclerosis caused by these strains. For example, neither the presence of circulating mediators of inflammation or aortic expression of TLR2 and 4 correlate with atherosclerosis in W83 infected animals [26]. Animals infected with A7436 do not exhibit an increase in circulating cytokines until infection is chronic (24 weeks or later), which coincides with a significant accumulation of lipid and foamy macrophages within the atheromatous plaque [24].

This study did not fully encompass the variety of *P. gingivalis* strains that have been reported to be associated with cardiovascular disease [40], [61], [70] such as *P. gingivalis* fimA type II strains, which are commonly found in human aortic aneurysms and atheromatous plaques [1], [40]. Nevertheless, we identified unique microbial strain specific interactions with endothelial cells that are likely to impact the manner in which this organism contributes to cardiovascular disease and likely other diseases as well. Our results underscore the necessity to consider that the mechanism by which one strain causes disease is not necessarily the same as other strains of *P. gingivalis*.

## Materials and Methods

### Cell culture

HCAE cells were obtained from Lonza,Walkersville, MD. Cell cultures were maintained with EBM®- 2 plus SingleQuots® medium (Lonza,Walkersville, MD) at 37°C/5% CO_2_ for all experiments. Only HCAE cells that underwent 8 or less passages were used for all experiments. For all experiments, cells were maintained at subconfluent conditions (≤90%).

### Bacterial strains and growth conditions

*P. gingivalis* strains used in this study included W83, 381 (from SUNY-Buffalo collection, Buffalo, NY), A7436 (a gift from Dr. Offenbacher, University of North Carolina Chapel Hill), and 33277 (American Type Culture Collection, Manassas, VA). All strains were cultured on supplemented blood agar plates or in tryptic soy broth (sTSB) supplemented with 5 µg of hemin and 1 µg vitamin K_1_ mL^−1^ as previously described [71]. Bacterial cultures were maintained in a Coy anaerobic chamber (Ann Arbor, MI) at an atmosphere of 10% H_2_, 5% CO_2_, and 85% N_2_.

For all HCAE cell infection experiments, bacterial cultures were grown to early stationary phase (approximately 18 hours) in sTSB. Bacterial numbers in sTSB cultures were determined by optical density readings performed at 550 nm. Inocula were prepared by dilution of the appropriate volume of sTSB culture in antibiotic free EBM-2 media to achieve a multiplicity of infection of 100. For all experiments, the final bacterial concentration of all inocula was confirmed by culture.

### Adherence Assays

Prior to inoculation, HCAE cells were washed 3 times with antibiotic free EBM-2 after which both the bacterial inocula and HCAE cells were chilled on ice for 15 minutes. Inoculated cells were then incubated at 4°C for 30 min without agitation. At time of harvest, cells were washed twice with ice cold EBM-2 and prepared for analysis by quantitative PCR (qPCR) or ELISA.

For qPCR assays, genomic DNA from HCAE cell lysates was extracted using the Wizard Genomic DNA Purification Kit (Promega, Madison, WI) according to manufacturer's instructions. Measurement of *P. gingivalis* 16S rDNA was performed with forward primer: 5′-CATAGATATCACGAGGAACTCCGATT, and reverse primer 5′ – AAACTGTTAGCAACTACCGATGTGG. For normalization purposes, eukaryotic 18S rDNA was measured with forward primer 5′- CGCCGCTAGAGGTGAAATTCT and reverse 5′ – CGAACCTCCGACTTTCGTTCT. QPCR reactions were performed using iQ™ SYBR® Green Supermix according to manufacturer's instructions (BioRad Laboratories, Hercules, CA) using an iCycler-IQ, version 3.1 using Optical System Software 3.1 (BioRad Laboratories, Hercules, CA). QPCR reactions were performed with the following thermocycler conditions: 40 cycles at 95°C for 3 min and 60°C for 45 seconds. Standard curves that ranged from 10^1^ to 10^7^ copies of 16S or 18S were used to calculate copy number per sample. 16S copy number was normalized to HCAE cell density, by dividing the 16S *P. gingivalis* copy number of each sample by its corresponding HCAE cell 18S copy number.

Quantification of *P. gingivalis* adherence by ELISA was performed as previously described [72], [73]. *P. gingivalis* was detected with a rabbit polyclonal antibody that was produced by immunization with whole cells of *P. gingivalis* strain W83 [Lot number C7947 – produced by Strategic Biosolutions, Newark, DE]. This antibody was determined to have the same avidity for *P. gingivalis* strains A7436, 381, and 33277. Briefly, HCAE cells were washed and fixed with 5% formalin in phosphate buffered saline (PBS) for 15 minutes at 37°C/5% CO_2_. Fixed cells were washed 3 times in PBS, prior to incubation with blocking buffer [5% bovine serum albumin, 2% goat serum, 0.1% Tween 20 in PBS] for 1 h at room temperature. After blocking, cells were incubated with anti-*P. gingivalis* antibody diluted 1∶5000 in blocking buffer, for one hour at room temperature. After washing with PBS-T (PBS containing 0.1% Tween), cells were probed with peroxidase-conjugated goat anti-rabbit IgG (MP Biomedicals Inc., Solon, OH), which was diluted 1∶6000 in blocking buffer. After 1 h at room temperature, cells were washed 4 times with PBS-T. After washing, cells were incubated with 3, 3′, 5, 5′ tetramethylbenzidine liquid substrate (Sigma-Aldrich, Inc., St. Louis, MO) for 5 min at room temperature. The reaction was stopped by the addition of 1 N HCl and color development was measured at 450 nm (Benchmark, Microplate reader, Bio-Rad Laboratories).

### Invasion Assays

HCAEC cells were washed with pre-warmed antibiotic free EBM-2 immediately before inoculation with *P. gingivalis* suspensions that were prepared as already described. Inoculated cells were incubated at 37°C/5% CO_2_ for 1.5 h. At the end of the incubation period, cells were washed with EBM-2 media and pulse treated [74] with antibiotic rich EBM-2 media [300 µg/ml gentamycin and 200 µg/ml metronidazole] (Sigma-Aldrich) for 1 h at 37°C/5% CO_2_ in order to kill any bacteria that were not internalized. At the end of the incubation period, cells were washed with antibiotic free EBM-2 and lysed by incubation in sterile water for 20 min at 37°C/5% CO_2_. Cell lysates were serially diluted 10 fold in sterile PBS and cultured on supplemented blood agar plates for the enumeration of bacteria.

Invasion assays with inhibition of autophagy were performed as already described with the following modifications. HCAE cells were pre-incubated with regular EBM-2 or 10 mM 3-MA (Sigma-Aldrich) dissolved in antibiotic free EBM-2 for 1 h before inoculation with *P. gingivalis.* Treatment with 3-MA was maintained during pulse antibiotic treatment and thereafter until 6 h post-inoculation.

### Persistence Assays

Persistence of *P. gingivalis* in HCAE cell cultures were inoculated and treated as already described. However, after pulse treatment with antibiotic rich media, cell cultures were washed again with antibiotic free EBM-2 and maintained in antibiotic free EBM-2 at 37°C/5% CO_2_ for 24 or 48 h. At time of harvest, supernatants were collected for microbial culture. HCAE cells were then lysed with sterile water and both HCAE cell supernatants and lysates were cultured for the presence of viable bacteria as described.

### Intracellular trafficking of bacteria

For transmission electron microscopy studies, HCAE cell cultures were sham inoculated or inoculated with *P. gingivalis* as already described. Inoculated cells were maintained undisturbed at 37°C/5% CO_2_ for 6 h before one hour fixation performed with 2% glutaraldehyde in PBS at room temperature. Fixed cells were processed for CMPase cytochemistry and imaged as previously described [36].

For colocalization studies with light chain three (LC3), HCAE cells were seeded onto sterile glass coverslips and transduced with GFP tagged-LC3 or GFP vectors packaged into an adenovirus (Welgen, Inc. Worcester, MA). Viruses were used at an MOI of 10. After 48 h, cells were inoculated with *P. gingivalis* as described and maintained undisturbed at 37°C/5% CO_2_. Uninfected cells were maintained with EBM-2 media (fed, negative control) or Krebs-Henseleit buffer (starved, positive control). HCAE cells were treated with 3-MA as already described for experiments involving inhibition of autophagy. At 6 h post-inoculation, HCAE cells were fixed with 4% paraformaldehyde dissolved in phosphate buffered saline (PBS) overnight at 4°C. Fixed cells were washed three times with PBS before mounting with ProLong® Gold Antifade reagent with DAPI (Invitrogen™). In experiments in which LAMP-1 was also detected, fixed cells were washed in PBS and incubated in a blocking solution (2% goat serum, 1% bovine serum albumin, 0.1% Triton X-100, 0.05% Tween 20, and 0.05% sodium azide in 0.01 M PBS) for 30 min at room temperature. Cells were then incubated overnight at 4°C with rabbit polyclonal anti-LAMP-1 antibody (Abcam®, Cambridge, MA), which was diluted 1∶200 in blocking buffer. Incubated coverslips were washed in PBS, and then incubated with ALEXA 594 goat-anti-rabbit (Life Technologies™) diluted 1∶2000 in blocking buffer, for 30 min at room temperature. After washing, cover slips were mounted in the manner described above. HCAE cells were visualized with an Olympus DSU-IX81 Spinning Disc Confocal microscope. At least five images for each sample were captured with Slidebook software (Olympus, Center Valley, PA). For publication purposes, final processing of images was performed with Image J software (US National Institutes of Health, Bethesda, MD) and Adobe Photoshop.

### Detection of pro-inflammatory cytokines, chemokines, and soluble cell adhesion molecules

Supernatants from HCAE cell invasion assays collected 24 h post-inoculation were evaluated for soluble adhesion molecules using the Milliplex detection kits (Millipore, St. Charles, MO). Culture supernatants and soluble mediator capture-bead-cocktails were incubated overnight at 4°C, washed, and then incubated with biotin labeled anti- cytokine for 1.5 h, followed by 30 min incubation with 1∶12.5 dilution of SAV at RT in the dark while gently shaking. Data were acquired using a Luminex®100™ and analyzed using Milliplex Analyst software (Viagene), standard curves and five-parameter logistics.

### HCAE cell viability

HCAE cells were seeded onto sterile coverslips and inoculated with the bacteria at an MOI of 100∶1 for 24 and 48 h, after which cell viability was evaluated with the LIVE/DEAD® Viability/Cytotoxicity Kit (Invitrogen Inc, Carlsbad, CA) according to the manufacturer's instructions. Briefly, supernatants were removed and cells were washed with PBS, stained with 2 µM of the polyanionic dye calcein AM and 4 µM of the plasma membrane excluded ethidium homodimer-1 (EthD-1) and evaluated by fluorescence microscopy. Live cells were detected with a fluorescein optical filter. Dead cells were detected with a rhodamine red filter. Five images from each sample were randomly acquired at 200× magnification with a Leica DM IRBE microscope. Dead and live cells within each image were counted, and at least 5 images per sample were examined. Percent mortality was calculated by dividing the total number of dead cells by the total number of live cells per sample.

### Statistical analysis

Statistical analyses were performed using GraphPad Prism software. ANOVA and Tukey's test were used for comparisons among experimental groups. An unpaired student's *t* test was used for comparisons that were limited to two groups. For all analyses a probability of *P*<0.05 was considered significant.

## Supporting Information

Figure S1 **FimA genotyping of encapsulated (A) and unencapsulated (B) strains of** ***P. gingivalis*** **.** *P. gingivalis* cultures were grown to late log phase as described in methods. Genomic DNA was extracted with Promega Wizard Genomic DNA Purification Kit (Madison, WI). PCR based genotyping was performed with published primer sets as already described [39].
(PDF)

Figure S2 **Ad-GFP-LC3 vector (A) and LAMP-1 isotype (B) controls.** A) HCAE cells were transduced with either Ad-GFP or AD-GFP-LC3 (MOI 10). At 48 hours post-transduction, cells were starved by incubating them in Krebs-Henseleit buffer. B) HCAE cells inoculated with *P. gingivalis* A7436. After 6 hours, cells were fixed with 4% paraformaldehyde dissolved in phosphate buffered saline (PBS) overnight at 4°C and processed as described in the methods section.
(PDF)

Figure S3 **Representative microscopic images of fed uninfected HCAE cells (HCAEC) and HCAE cells infected with** ***P. gingivalis*** **strains A7436, 381, and 33277 within LC3 positive vacuoles.** Cells were processed at 6 hours post-inoculation. Arrows indicate bacteria within LC3 positive vacuoles. Scale bar is equivalent to 10 µm.
(PDF)

Figure S4 **Representative microscopic images of** ***P. gingivalis*** **strains A7436, 381, and 33277 within LAMP-1 positive vacuoles at 6 hours post-inoculation.** Arrows indicate bacteria within LAMP-1 positive vacuoles. Scale bar is equivalent to 10 µm.
(PDF)

Figure S5 **Inhibition of autophagy with 3-MA.** At 48 hours post-transduction with Ad-GFP-LC3 (MOI 10), transduced cells infected were pre-treated with 10 mM 3-MA one hour prior to infection with P. gingivalis 381, which was added at an MOI of 100. Treatment with 3-MA was maintained in infected cultures until time of harvest (6 hours post inoculation). Harvested cells were processed and imaged as described in the methods section.
(PDF)

Figure S6 **Assessment of HCAE cell viability and toxicity after infection with** ***P. gingivalis.*** Representative images of uninfected (A) and *P. gingivalis* strain W83 infected (B) HCAE cells at 24 hours post-inoculation. Images were obtained at 200× magnification (scale bar = 100 µm). Cell viability was evaluated by esterase mediated conversion of calcein-AM to calcein (green). Loss of membrane permeability was measured by uptake of ethidium homodimer-1 (red). (C) Mean percent ± SD (n = 2) of HCAE cell death at 24 and 48 hours post-inoculation. Values represent results from two independent experiments. Percent mortality was calculated by dividing the total number of dead cells by the total number of live cells per sample. Live cells were detected with a fluorescein optical filter. Dead cells were detected with a rhodamine red filter. Images from each sample were randomly acquired at 200× magnification with a Leica DM IRBE microscope. Dead and live cells within each image were counted, and at least 5 images per sample were examined.
(PDF)
