# Supplementary material for: Porphyromonas gingivalis Strain Specific Interactions with Human Coronary Artery Endothelial Cells: A Comparative Study
Source: PLoS One. 2012 Dec 26;7(12):e52606. doi: 10.1371/journal.pone.0052606 (PMC3530483; doi:10.1371/journal.pone.0052606)
Supplement: Figure S1 — FimA genotyping of encapsulated (A) and unencapsulated (B) strains of P. gingivalis . P. gingivalis cultures were grown to late log phase as described in methods. Genomic DNA was extracted with Promega Wizard Genomic DNA Purification Kit (Madison, WI). PCR based genotyping was performed with published primer sets as already described [39]. (PDF) [file pone.0052606.s001.pdf]

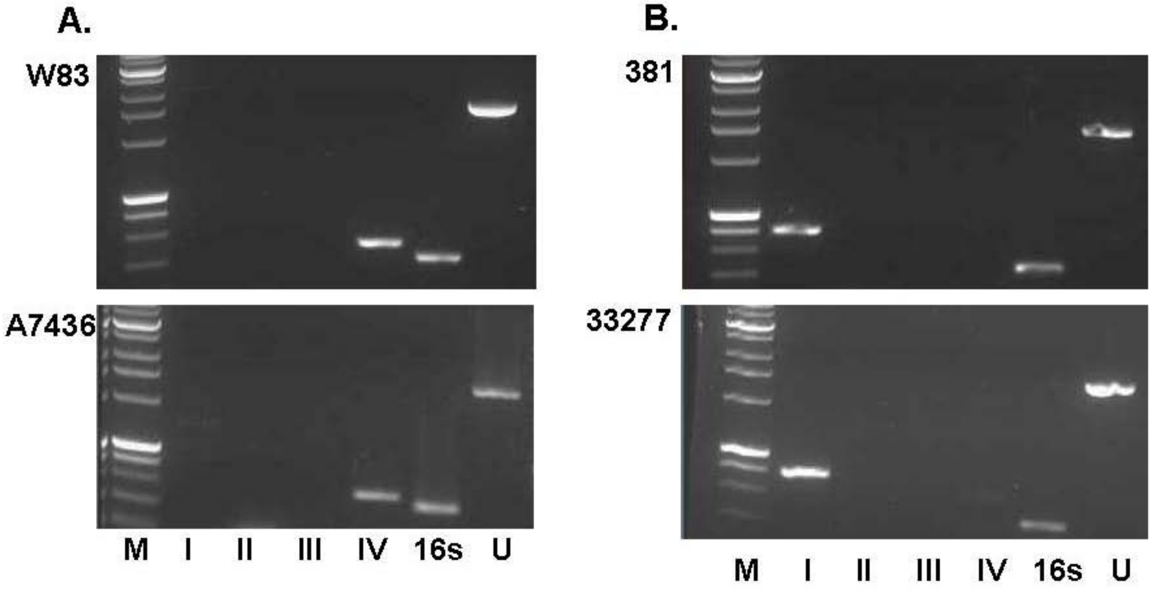

Figure S1. FimA genotyping of encapsulated (A) and unencapsulated (B) strains of *P. gingivalis* *P. gingivalis* cultures were grown to late log phase as described in methods. Genomic DNA was extracted with Promega Wizard Genomic DNA Purification Kit (Madison, WI). PCR based genotyping was performed with published primer sets as already described [39].
