# Supplementary material for: Porphyromonas gingivalis Strain Specific Interactions with Human Coronary Artery Endothelial Cells: A Comparative Study
Source: PLoS One. 2012 Dec 26;7(12):e52606. doi: 10.1371/journal.pone.0052606 (PMC3530483; doi:10.1371/journal.pone.0052606)
Supplement: Figure S2 — Ad-GFP-LC3 vector (A) and LAMP-1 isotype (B) controls. A) HCAE cells were transduced with either Ad-GFP or AD-GFP-LC3 (MOI 10). At 48 hours post-transduction, cells were starved by incubating them in Krebs-Henseleit buffer. B) HCAE cells inoculated with P. gingivalis A7436. After 6 hours, cells were fixed with 4% paraformaldehyde dissolved in phosphate buffered saline (PBS) overnight at 4°C and processed as described in the methods section. (PDF) [file pone.0052606.s002.pdf]

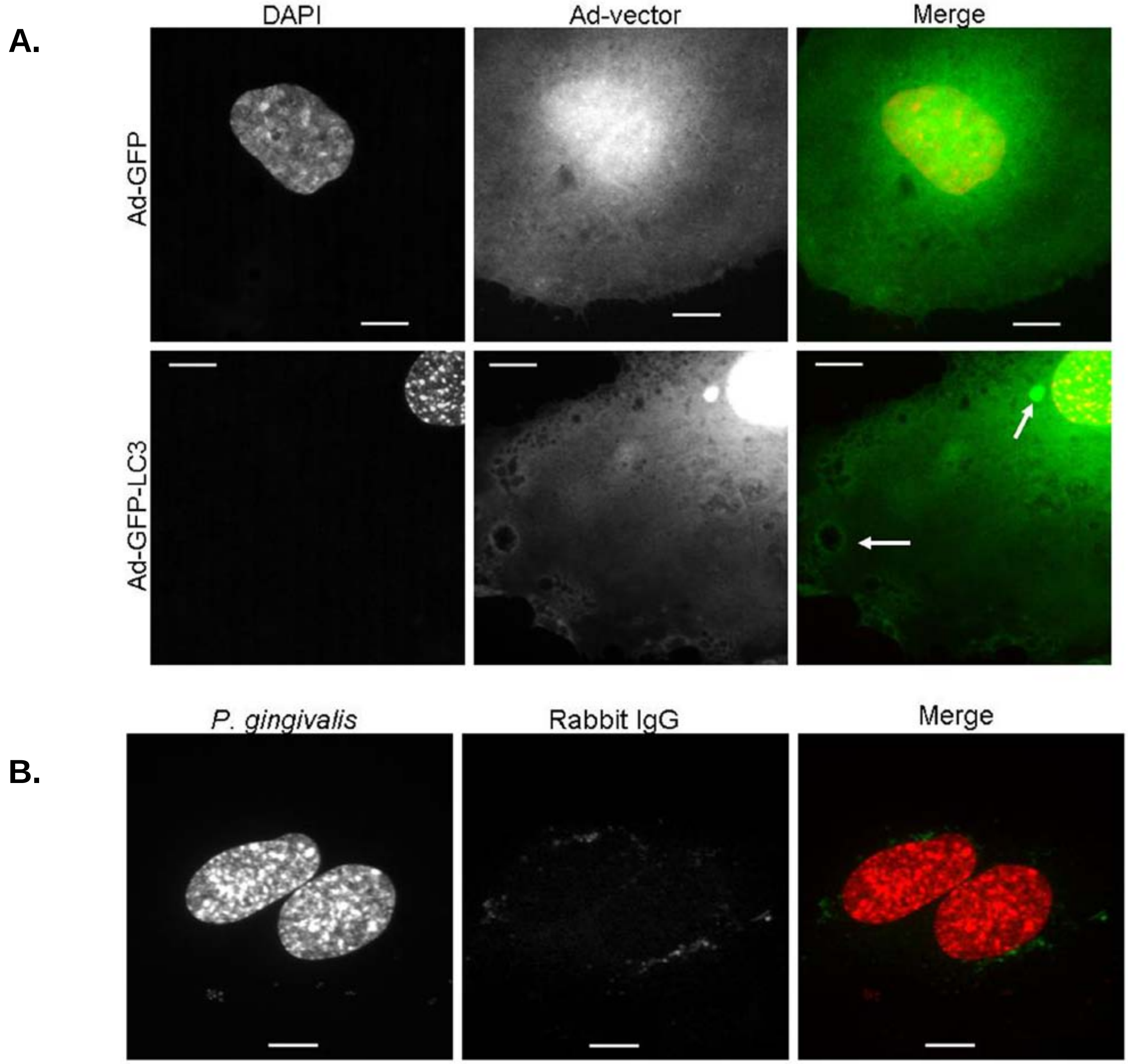

Figure S2. Ad-GFP-LC3 vector (A) and LAMP-1 isotype (B) controls.
A) HCAE cells were transduced with either Ad-GFP or AD-GFP-LC3 (MOI 10). At 48 hours post-transduction, cells were starved by incubating them in Krebs-Henseleit buffer. B) HCAE cells inoculated with *P. gingivalis* A7436. After 6 hours, cells were fixed with 4% paraformaldehyde dissolved in phosphate buffered saline (PBS) overnight at 4^{0}C and processed as described in the methods section.
