# Supplementary material for: Porphyromonas gingivalis Strain Specific Interactions with Human Coronary Artery Endothelial Cells: A Comparative Study
Source: PLoS One. 2012 Dec 26;7(12):e52606. doi: 10.1371/journal.pone.0052606 (PMC3530483; doi:10.1371/journal.pone.0052606)
Supplement: Figure S3 — Representative microscopic images of fed uninfected HCAE cells (HCAEC) and HCAE cells infected with P. gingivalis strains A7436, 381, and 33277 within LC3 positive vacuoles. Cells were processed at 6 hours post-inoculation. Arrows indicate bacteria within LC3 positive vacuoles. Scale bar is equivalent to 10 µm. (PDF) [file pone.0052606.s003.pdf]

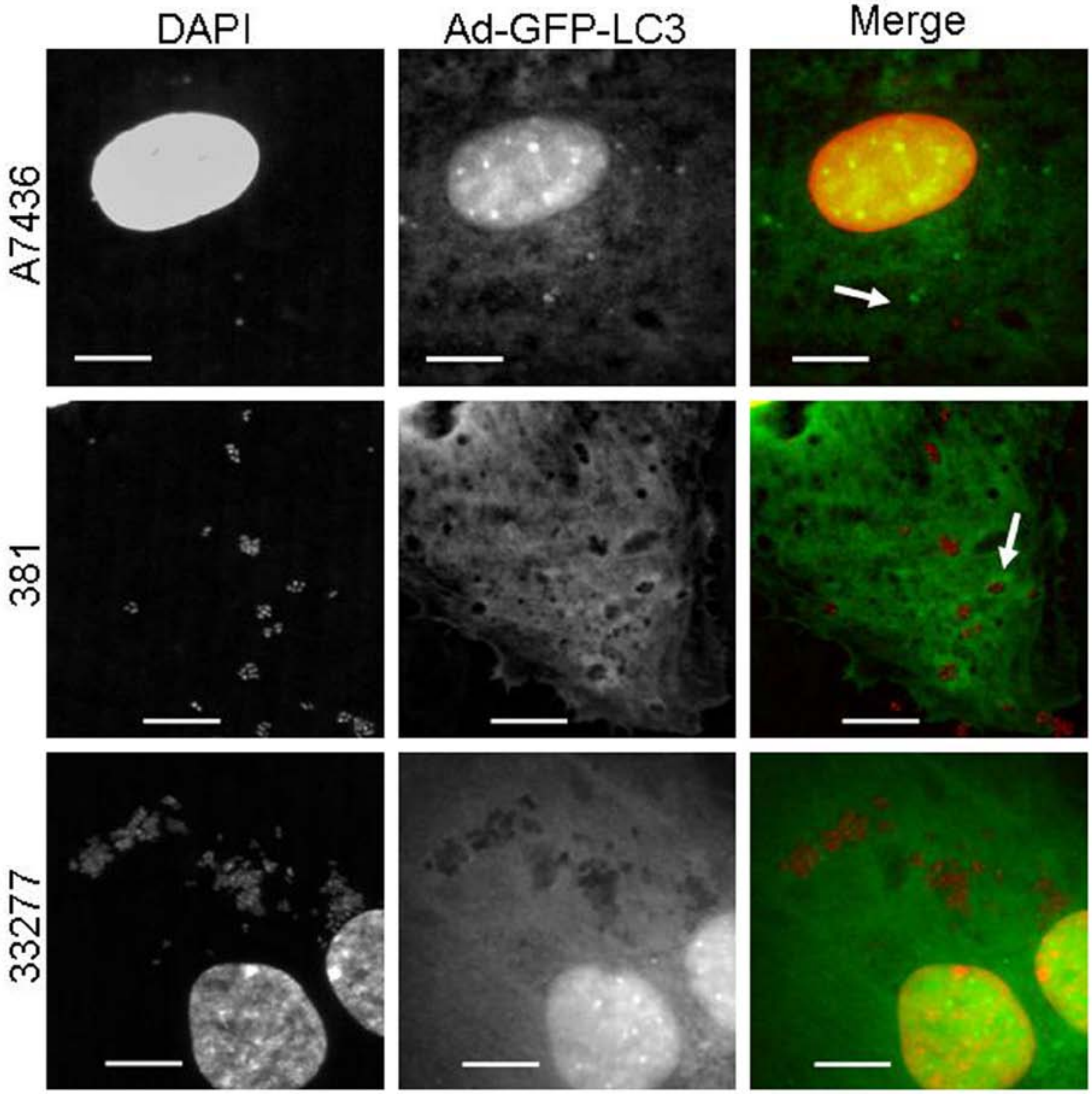

Figure S3. Representative microscopic images of *P. gingivalis* strains A7436, 381, and 33277 within LC3 positive vacuoles. Cells were processed at 6 hours post-inoculation. Arrows indicate bacteria within LC3 positive vacuoles. Scale bar is equivalent to 10 µm.
