# Supplementary material for: Porphyromonas gingivalis Strain Specific Interactions with Human Coronary Artery Endothelial Cells: A Comparative Study
Source: PLoS One. 2012 Dec 26;7(12):e52606. doi: 10.1371/journal.pone.0052606 (PMC3530483; doi:10.1371/journal.pone.0052606)
Supplement: Figure S4 — Representative microscopic images of P. gingivalis strains A7436, 381, and 33277 within LAMP-1 positive vacuoles at 6 hours post-inoculation. Arrows indicate bacteria within LAMP-1 positive vacuoles. Scale bar is equivalent to 10 µm. (PDF) [file pone.0052606.s004.pdf]

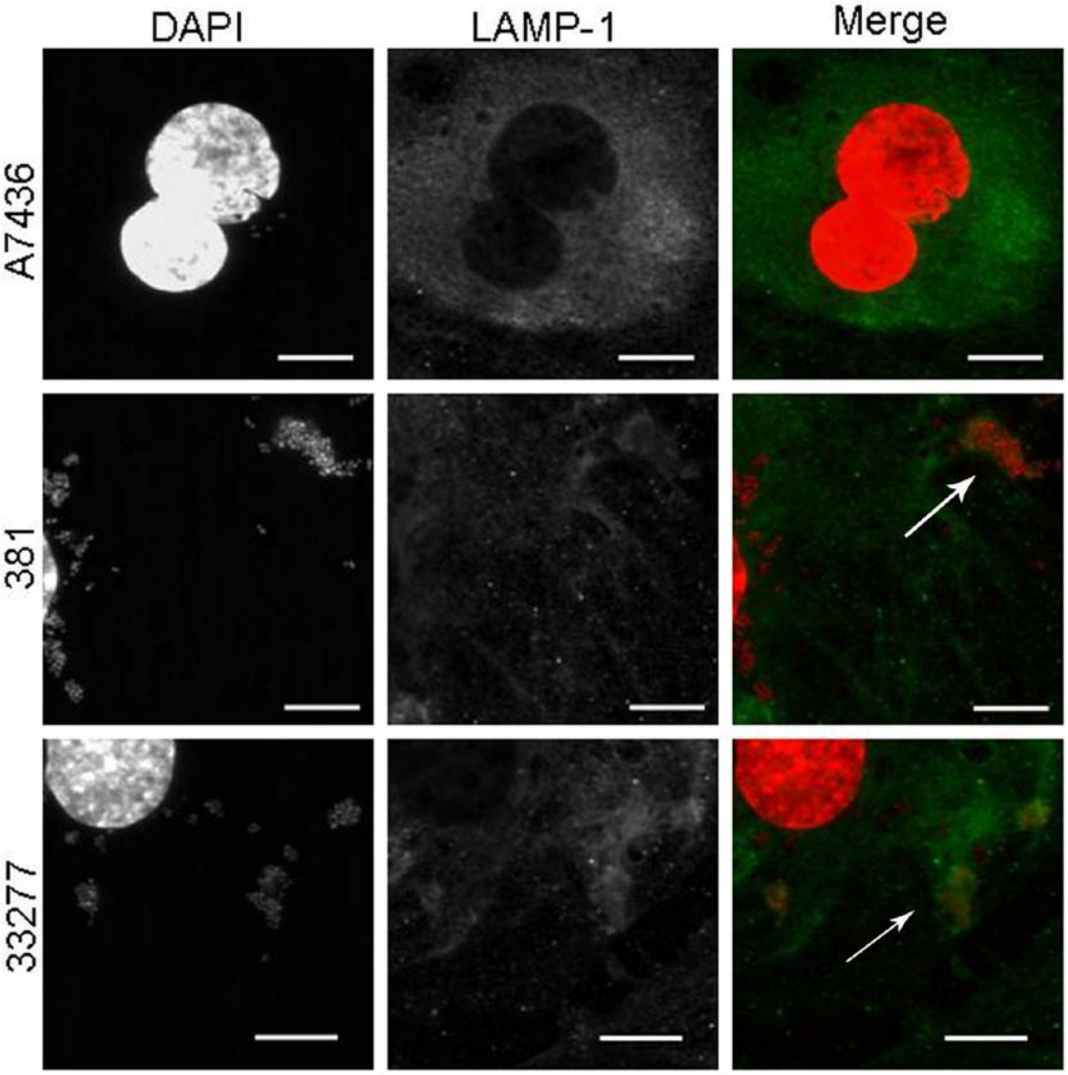

Figure S4. Representative microscopic images of *P. gingivalis* strains A7436, 381, and 33277 within LAMP-1 positive vacuoles at 6 hours post-inoculation. Arrows indicate bacteria within LAMP-1 positive vacuoles. Scale bar is equivalent to 10 μm.
