# Supplementary material for: Porphyromonas gingivalis Strain Specific Interactions with Human Coronary Artery Endothelial Cells: A Comparative Study
Source: PLoS One. 2012 Dec 26;7(12):e52606. doi: 10.1371/journal.pone.0052606 (PMC3530483; doi:10.1371/journal.pone.0052606)
Supplement: Figure S5 — Inhibition of autophagy with 3-MA. At 48 hours post-transduction with Ad-GFP-LC3 (MOI 10), transduced cells infected were pre-treated with 10 mM 3-MA one hour prior to infection with P. gingivalis 381, which was added at an MOI of 100. Treatment with 3-MA was maintained in infected cultures until time of harvest (6 hours post inoculation). Harvested cells were processed and imaged as described in the methods section. (PDF) [file pone.0052606.s005.pdf]

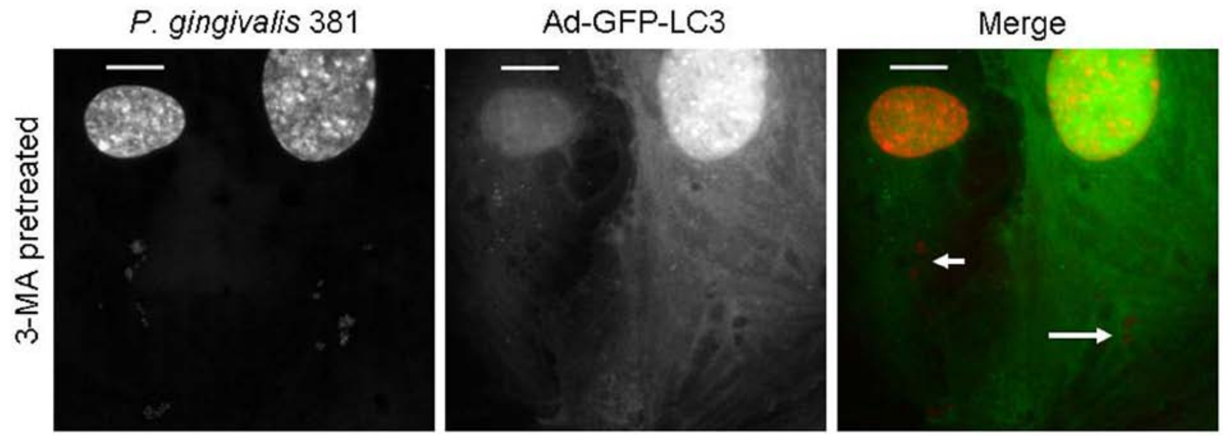

Figure S5. Inhibition of autophagy with 3-MA. At 48 hours post-transduction with Ad-GFP-LC3 (MOI 10), transduced cells infected were pre-treated with 10 mM 3-MA one hour prior to infection with P. gingivalis 381, which was added at an MOI of 100. Treatment with 3-MA was maintained in infected cultures until time of harvest (6 hours post inoculation). Harvested cells were processed and imaged as described in the methods section.
