# Supplementary material for: Porphyromonas gingivalis Strain Specific Interactions with Human Coronary Artery Endothelial Cells: A Comparative Study
Source: PLoS One. 2012 Dec 26;7(12):e52606. doi: 10.1371/journal.pone.0052606 (PMC3530483; doi:10.1371/journal.pone.0052606)
Supplement: Figure S6 — Assessment of HCAE cell viability and toxicity after infection with P. gingivalis. Representative images of uninfected (A) and P. gingivalis strain W83 infected (B) HCAE cells at 24 hours post-inoculation. Images were obtained at 200× magnification (scale bar = 100 µm). Cell viability was evaluated by esterase mediated conversion of calcein-AM to calcein (green). Loss of membrane permeability was measured by uptake of ethidium homodimer-1 (red). (C) Mean percent ± SD (n = 2) of HCAE cell death at 24 and 48 hours post-inoculation. Values represent results from two independent experiments. Percent mortality was calculated by dividing the total number of dead cells by the total number of live cells per sample. Live cells were detected with a fluorescein optical filter. Dead cells were detected with a rhodamine red filter. Images from each sample were randomly acquired at 200× magnification with a Leica DM IRBE microscope. Dead and live cells within each image were counted, and at least 5 images per sample were examined. (PDF) [file pone.0052606.s006.pdf]

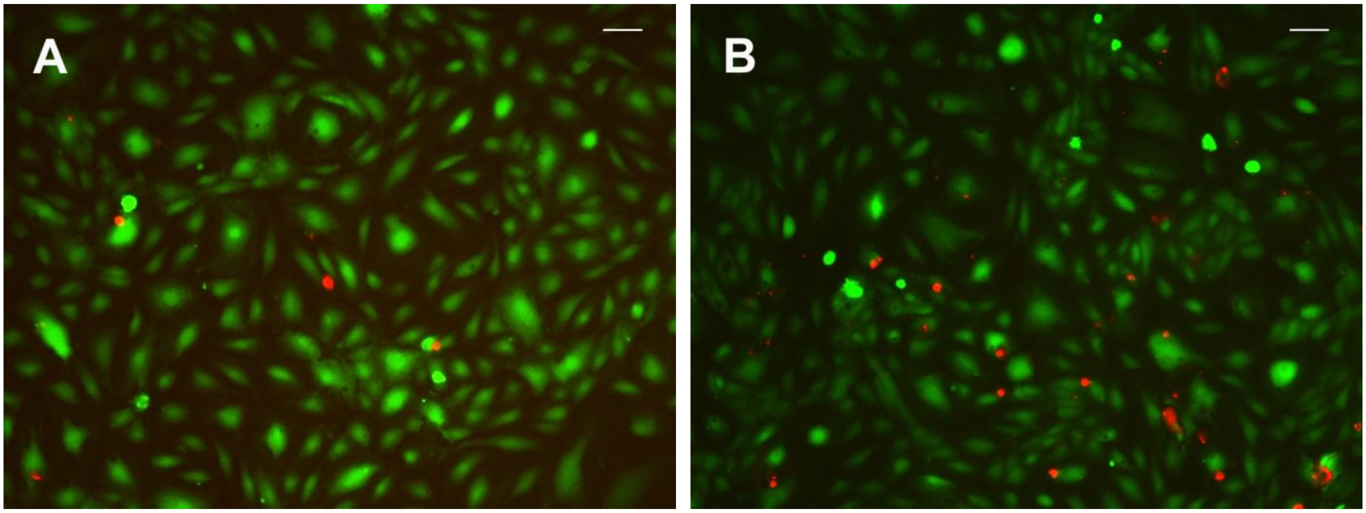

C.

	% mortality (mean ±SD)	
**Treatment**	**24 hrs PI**	**48 hrs PI**
Control	2.19 ± 1	6.88 ± 2
W83	4.96 ± 3	6.05 ± 2
A7436	2.43 ± 1	3.34 ± 1
381	9.55 ± 7	8.00 ± 2
33277	4.52 ± 3	3.40 ± 1

Figure S6. Assessment of HCAE cell viability and toxicity after infection with *P. gingivalis.* Representative images of uninfected (A) and *P. gingivalis* strain W83 infected (B) HCAE cells at 24 hours post-inoculation. Images were obtained at 200x magnification (scale bar = 100µm). Cell viability was evaluated by esterase mediated conversion of calcein-AM to calcein (green). Loss of membrane permeability was measured by uptake of ethidium homodimer-1 (red). (C) Mean percent ± SD (n = 2) of HCAE cell death at 24 and 48 hours post-inoculation. Values represent results from two independent experiments. Percent mortality was calculated by dividing the total number of dead cells by the total number of live cells per sample. Live cells were detected with a fluorescein optical filter. Dead cells were detected with a rhodamine red filter. Images from each sample were randomly acquired at 200x magnification with a Leica DM IRBE microscope. Dead and live cells within each image were counted, and at least 5 images per sample were examined.
